# Supplementary material for: Akaluc/AkaLumine bioluminescence system enables highly sensitive, non-invasive and temporal monitoring of gene expression in Drosophila
Source: Commun Biol. 2023 Dec 14;6:1270. doi: 10.1038/s42003-023-05628-x (PMC10721803; doi:10.1038/s42003-023-05628-x)
Supplement: Supplementary file 2 — Supplementary Information [file 42003_2023_5628_MOESM2_ESM.docx]

**Supplementary Information**

**Akaluc/AkaLumine bioluminescence system enables highly sensitive, non-invasive and temporal monitoring of gene expression in *Drosophila***

Akira Ito^1^, Nagisa Matsuda^1^, Yumiko Ukita^1^, Misako Okumura^1,2^, Takahiro Chihara^1,2,*^

^1^Program of Biomedical Science, Graduate School of Integrated Sciences for Life, Hiroshima University, 1-3-1 Kagamiyama, Higashi-Hiroshima, Hiroshima, Japan

^2^Program of Basic Biology, Graduate School of Integrated Sciences for Life, Hiroshima University, 1-3-1 Kagamiyama, Higashi-Hiroshima, Hiroshima, Japan

*Corresponding author: Takahiro Chihara

E-mail: [tchihara@hiroshima-u.ac.jp](mailto:tchihara@hiroshima-u.ac.jp)


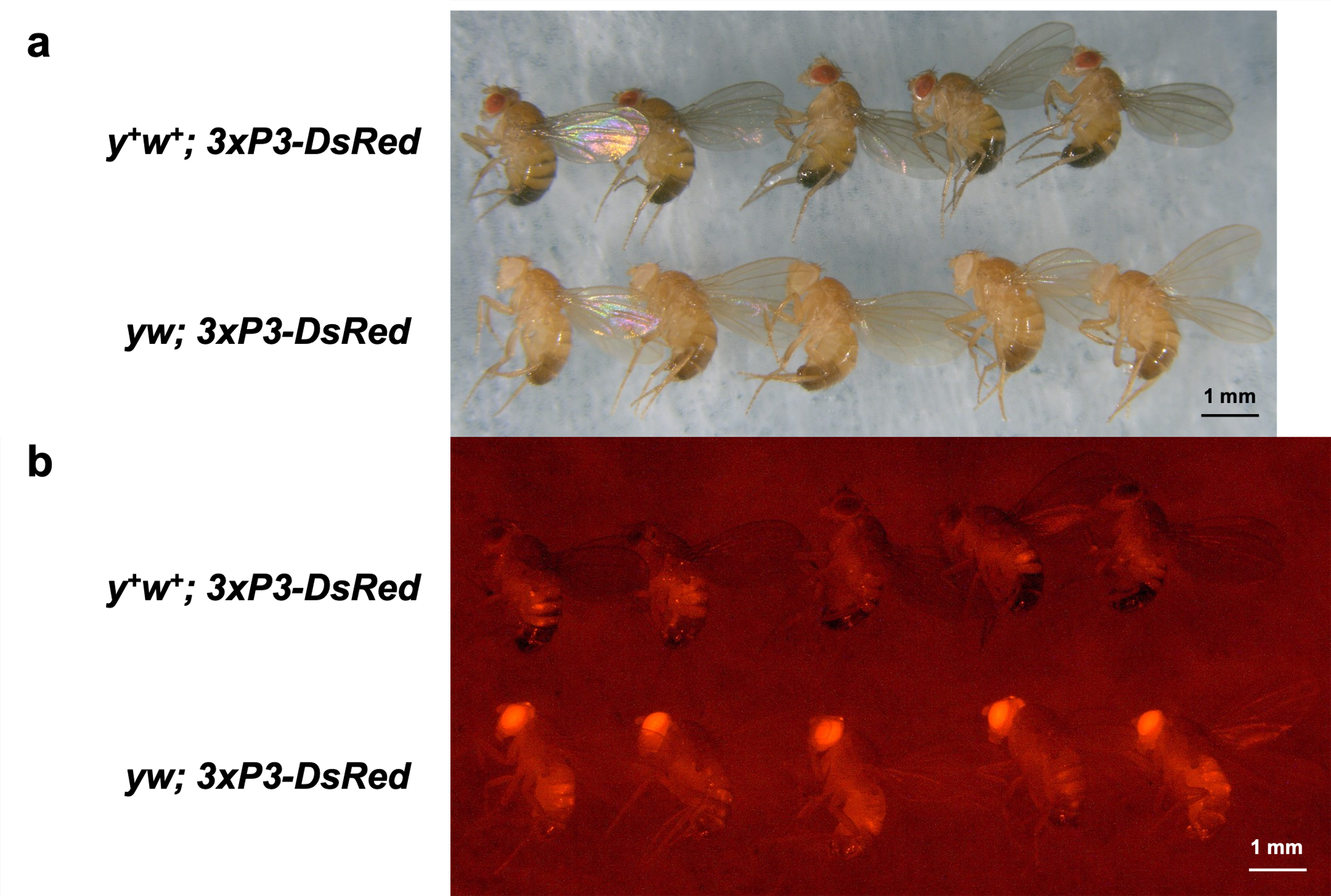


**Supplementary Fig. 1. Absorption and scattering of photons by pigments in *Drosophila***

(a, b) The presence or absence of the *white* (*w*) and *yellow* (*y*) genes involved in pigment synthesis significantly alter the DsRed signal. Photographs were taken with a fluorescence stereomicroscope (Leica M165 FC) and digital camera (Leica MC170 HD) in the bright field (a) and with ET DSR filter set (Excitation: 545/40, Barrier: 620/60) (b). In each photo, the upper fly is a strain with wild-type body pigment (*y^+^w^+^*), and the lower fly is a strain lacking eye and cuticle pigment (*y w*). Both strains express the same copy number of *DsRed* gene by the 3xP3 promoter. Scale bar at the bottom right of each photo indicates 1 mm.


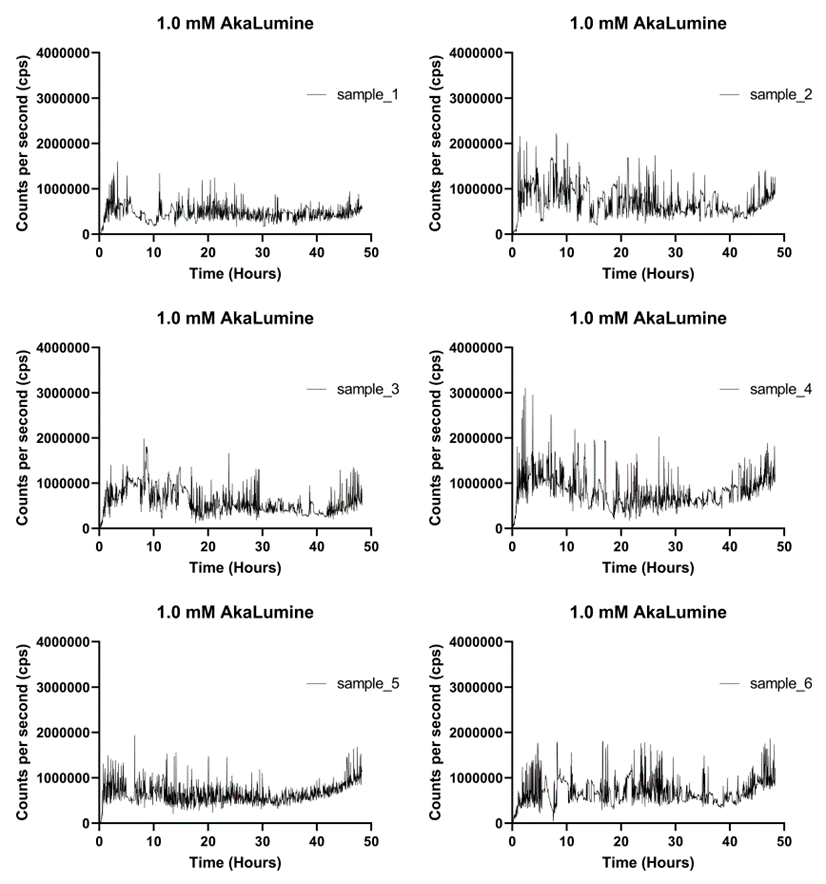


**Supplementary Fig. 2. Individual differences in luminescence when AkaLumine is administered to Akaluc-expressing flies**

There was no significant difference in luminescence changes after administration of AkaLumine to *tubP-Gal4>UAS-Venus-Akaluc* flies at the individual level. This figure shows the results of Figure 1d when 1.0 mM AkaLumine was administered separately to each individual. Luminescence measurements were started as soon as AkaLumine was administered to Akaluc-expressing flies, and this figure shows the results of each measurement until 48 h.


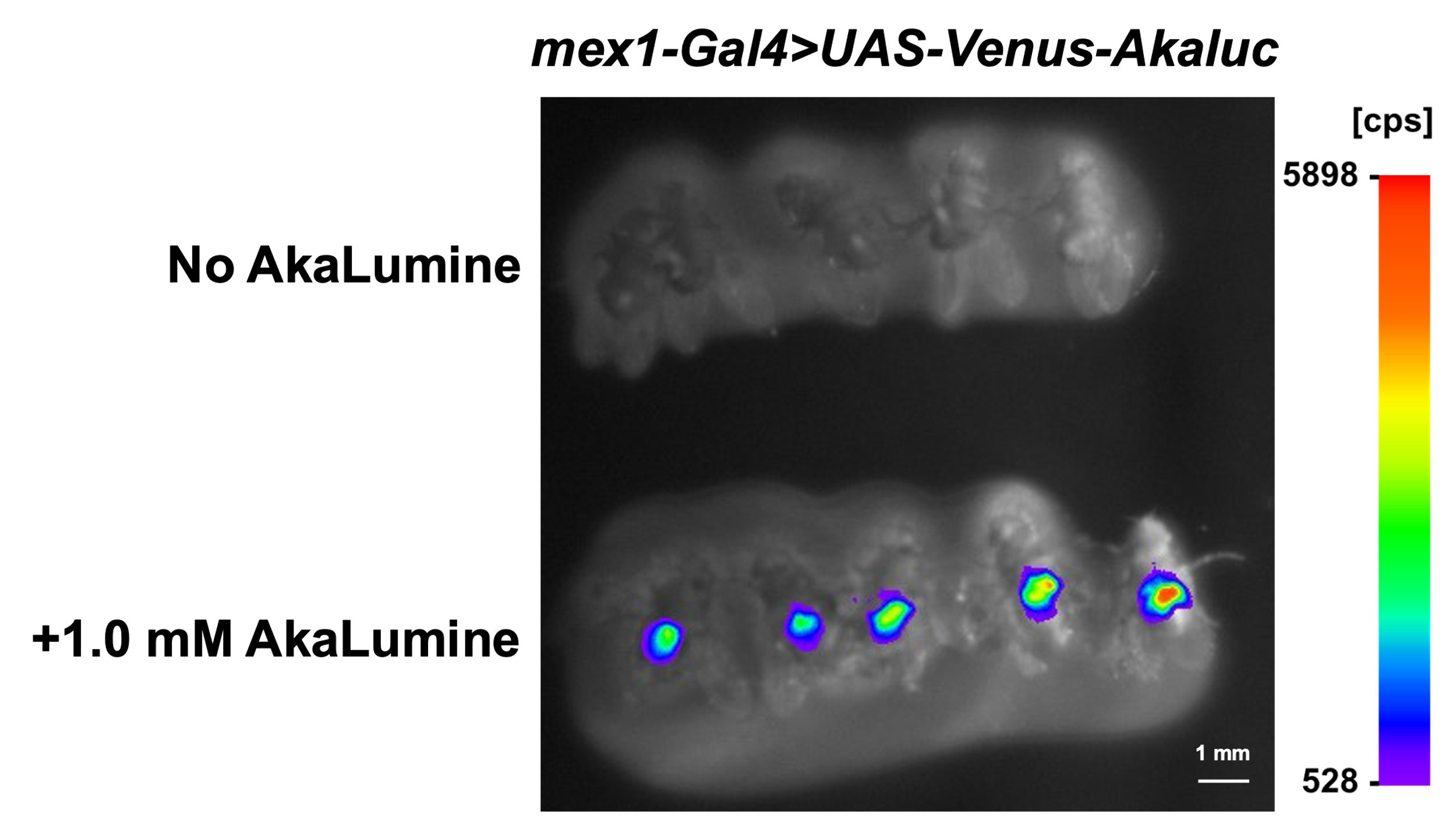


**Supplementary Fig. 3. Akaluc/AkaLumine imaging from the gut**

We examined the spatial resolution of Akaluc/AkaLumine bioluminescence imaging using a strain that expresses Akaluc in a gut-specific manner (*mex1-Gal4>UAS-Venus-Akaluc*). We found that the shape of the gut could not be recognized, but the signal could be detected only from the abdomen, where the gut is located. The AkaLumine-treated group was administered 1.0 mM AkaLumine for 24 hours, and luminescence imaging was performed (lower flies). The control group was fed normal food (upper flies). The scale bar in the bottom right of the photo indicates 1 mm.


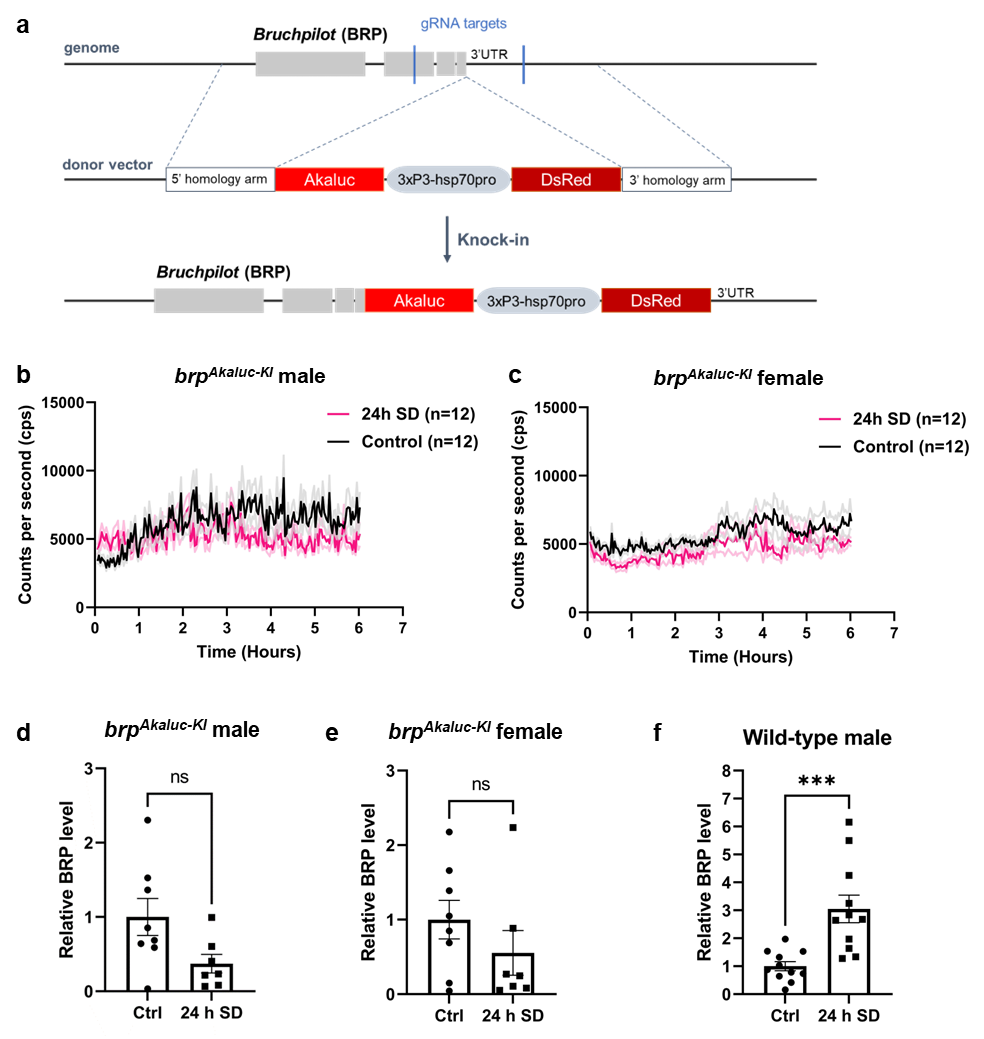


**Supplementary Fig. 4. The monitoring of the endogenous Brp protein level using *brp^Akaluc-KI^* strain**

In strains with Akaluc fused to endogenous Brp, no increase in Brp protein levels due to sleep deprivation was observed in both luminescence measurements and Western blotting results. (a) Schematic diagram of *brp^Akaluc-KI^* strain generation. Akaluc was inserted in-frame at the 3' end of the *Brp* coding region without the stop codon by homologous recombination using the CRISPR/Cas9 system. Along with Akaluc, DsRed was also introduced as a knock-in marker. (b, c) Luminescence measurements using the *brp^Akaluc-KI^* strain showed no increase in Brp signal due to 24 h sleep deprivation in both males (b) and females (c). *brp^Akaluc-KI^* flies were administered AkaLumine (1.0 mM) while being vibrated at a rate of 5 s min^-1^ using a vortex mixer for 24 h of sleep deprivation (24 h SD: magenta). The control group was not sleep-deprived (Control: black). Luminescence measurements were performed continuously for 6 h with AkaLumine (1.0 mM) administered to the flies. The darker-colored lines indicate the mean, and the lighter-colored lines indicate the error bars (s.e.m). n = 12. (d, e) In quantitative analysis of Brp levels by Western blotting using the *brp^Akaluc-KI^* strains, no increase in Brp protein levels was observed in either males (d) or females (e) due to 24 h sleep deprivation. Like the luminescence measurement experiment, the *brp^Akaluc-KI^* strains were sleep-deprived for 24 hours, and Western blotting was performed (24 h SD). The control group was not sleep-deprived (Ctrl). Unpaired t-test was used for statistical analysis. ns: no significance, n = 8 (Ctrl) and n =7 (24 h SD). Error bars indicate s.e.m. (f) The increase in Brp levels due to sleep deprivation was observed when wild-type flies were used, indicating that the sleep deprivation technique was not problematic. Male wild-type flies (*w^1118^*) were compared by Western blotting in a group with 24 h sleep deprivation (24 h SD) and a group without sleep deprivation (Ctrl) using the same method as above. Unpaired t-test was used for statistical analysis. ***p < 0.001, n = 11. Error bars indicate s.e.m.
