# Supplementary material for: Akaluc/AkaLumine bioluminescence system enables highly sensitive, non-invasive and temporal monitoring of gene expression in Drosophila
Source: Commun Biol. 2023 Dec 14;6:1270. doi: 10.1038/s42003-023-05628-x (PMC10721803; doi:10.1038/s42003-023-05628-x)
Supplement: Supplementary file 5 — Reporting Summary [file 42003_2023_5628_MOESM5_ESM.pdf]

nature portfolio

Corresponding author(s): Takahiro Chihara

Last updated by author(s): Nov 22, 2023

Reporting Summary

Nature Portfolio wishes to improve the reproducibility of the work that we publish. This form provides structure for consistency and transparency in reporting. For further information on Nature Portfolio policies, see our Editorial Policies and the Editorial Policy Checklist.

Statistics

For all statistical analyses, confirm that the following items are present in the figure legend, table legend, main text, or Methods section.

n/a	Confirmed	
☐	☒	The exact sample size (*n*) for each experimental group/condition, given as a discrete number and unit of measurement
☐	☒	A statement on whether measurements were taken from distinct samples or whether the same sample was measured repeatedly
☐	☒	The statistical test(s) used AND whether they are one- or two-sided<br>*Only common tests should be described solely by name; describe more complex techniques in the Methods section.*
☒	☐	A description of all covariates tested
☐	☒	A description of any assumptions or corrections, such as tests of normality and adjustment for multiple comparisons
☐	☒	A full description of the statistical parameters including central tendency (e.g. means) or other basic estimates (e.g. regression coefficient) AND variation (e.g. standard deviation) or associated estimates of uncertainty (e.g. confidence intervals)
☐	☒	For null hypothesis testing, the test statistic (e.g. *F*, *t*, *r*) with confidence intervals, effect sizes, degrees of freedom and *P* value noted<br>*Give P values as exact values whenever suitable.*
☒	☐	For Bayesian analysis, information on the choice of priors and Markov chain Monte Carlo settings
☒	☐	For hierarchical and complex designs, identification of the appropriate level for tests and full reporting of outcomes
☒	☐	Estimates of effect sizes (e.g. Cohen's *d*, Pearson's *r*), indicating how they were calculated

Our web collection on statistics for biologists contains articles on many of the points above.

Software and code

Policy information about availability of computer code

Data collection	No software were used to collect data.
Data analysis	Prism9 software (GraphPad) was used to analyze the data.

For manuscripts utilizing custom algorithms or software that are central to the research but not yet described in published literature, software must be made available to editors and reviewers. We strongly encourage code deposition in a community repository (e.g. GitHub). See the Nature Portfolio guidelines for submitting code & software for further information.

Data

Policy information about availability of data

All manuscripts must include a data availability statement. This statement should provide the following information, where applicable:

- Accession codes, unique identifiers, or web links for publicly available datasets
- A description of any restrictions on data availability
- For clinical datasets or third party data, please ensure that the statement adheres to our policy

Numerical source data supporting the results of this study are provided as 'Supplementary Data 1'.

Research involving human participants, their data, or biological material

Policy information about studies with human participants or human data. See also policy information about sex, gender (identity/presentation), and sexual orientation and race, ethnicity and racism.

Reporting on sex and gender	N/A
Reporting on race, ethnicity, or other socially relevant groupings	N/A
Population characteristics	N/A
Recruitment	N/A
Ethics oversight	N/A

Note that full information on the approval of the study protocol must also be provided in the manuscript.

Field-specific reporting

Please select the one below that is the best fit for your research. If you are not sure, read the appropriate sections before making your selection.

☒ Life sciences ☐ Behavioural & social sciences ☐ Ecological, evolutionary & environmental sciences

For a reference copy of the document with all sections, see nature.com/documents/nr-reporting-summary-flat.pdf

Life sciences study design

All studies must disclose on these points even when the disclosure is negative.

Sample size	The sample size was determined based on our experience, experimental design, and sufficient statistics.
Data exclusions	In luminescence measurements, data from individuals that were dead after the measurement were excluded from the analysis.
Replication	Luminescence measurement and imaging experiments were performed at least twice to make sure that similar results could be reproduced. Experiments for real-time PCR, Brp experiments and measurement of pupariation and eclosion rates were performed only once.
Randomization	Randomization was not used.
Blinding	Blinding was not used.

Reporting for specific materials, systems and methods

We require information from authors about some types of materials, experimental systems and methods used in many studies. Here, indicate whether each material, system or method listed is relevant to your study. If you are not sure if a list item applies to your research, read the appropriate section before selecting a response.

Materials & experimental systems

n/a	Involved in the study	
☐	☒	Antibodies
☒	☐	Eukaryotic cell lines
☒	☐	Palaeontology and archaeology
☐	☒	Animals and other organisms
☒	☐	Clinical data
☒	☐	Dual use research of concern
☒	☐	Plants

Methods

n/a	Involved in the study	
☒	☐	ChIP-seq
☒	☐	Flow cytometry
☒	☐	MRI-based neuroimaging

Antibodies

Antibodies used	mouse monoclonal antibody to Drosophila Bruchpilot (Developmental Studies Hybridoma Bank), mouse monoclonal antibody to human α-tubulin (Cedarlane, CLT9002), Peroxidase AffiniPure Donkey Anti-Mouse IgG (Jackson ImmunoResearch, 715-035-151)

Validation	All antibodies used in this study have been validated and detailed information can be found at the manufacturer's website listed below.<br><br>mouse monoclonal antibody to Drosophila Bruchpilot (Developmental Studies Hybridoma Bank), https://dshb.biology.uiowa.edu/nc82<br>mouse monoclonal antibody to human α-tubulin (Cedarlane, CLT9002), https://www.cedarlanelabs.com/Products/Detail/CLT9002<br>Peroxidase AffiniPure Donkey Anti-Mouse IgG (Jackson ImmunoResearch, 715-035-151), https://www.jacksonimmuno.com/catalog/products/715-035-151

Animals and other research organisms

Policy information about studies involving animals; ARRIVE guidelines recommended for reporting animal research, and Sex and Gender in Research

Laboratory animals	Adult of Drosophila melanogaster (3-5 days old) were used for all experiments and measurements.
Wild animals	Wild animals were not used.
Reporting on sex	Female flies were used in all experiments except Supplemental Figure 1 and 4.
Field-collected samples	Field-collected samples were not used.
Ethics oversight	Guideline of "Recombinant DNA Experiments, Animal Experiments and Using Pathogen" in Hiroshima University, Japan

Note that full information on the approval of the study protocol must also be provided in the manuscript.
